# Zika purified inactivated virus (ZPIV) vaccine reduced vertical transmission in pregnant immunocompetent mice

**DOI:** 10.1038/s41541-024-00823-1

**Published:** 2024-02-15

**Authors:** In-Jeong Kim, Michael P. Tighe, Paula A. Lanthier, Madeline J. Clark, Rafael A. De La Barrera, Vincent Dussupt, Letzibeth Mendez-Rivera, Shelly J. Krebs, Kelsey L. Travis, Timothy C. Low-Beer, Tres S. Cookenham, Kathleen G. Lanzer, Derek T. Bernacki, Frank M. Szaba, Amanda A. Schneck, Jerrold Ward, Stephen J. Thomas, Kayvon Modjarrad, Marcia A. Blackman

**Affiliations:** 1 Trudeau Institute, Inc., Saranac Lake, NY 12983 USA; 2 Pilot Bioproduction Facility, Center for Enabling Capabilities, Walter Reed Army Institute of Research, Silver Spring, MD 20910 USA; 3 Emerging Infectious Diseases Branch, Walter Reed Army Institute of Research, Silver Spring, MD 20910 USA; 4 U.S. Military HIV Research Program, Center of Infectious Disease Research, Walter Reed Army Institute of Research, Silver Spring, MD 20910 USA; 5 Henry M. Jackson Foundation for the Advancement of Military Medicine, Bethesda, MD 20817 USA; 6 Global VetPathology, Montgomery Village, MD 20886 USA; 7 Institute for Global Health and Translational Sciences, State University of New York, Upstate Medical University, Syracuse, NY 13210 USA; 8 Present Address: Pfizer Inc. Vaccine Research and Development, Pearl River, NY 10965 USA

**Keywords:** Inactivated vaccines, Viral infection

## Abstract

Zika virus (ZIKV) is a significant threat to pregnant women and their fetuses as it can cause severe birth defects and congenital neurodevelopmental disorders, referred to as congenital Zika syndrome (CZS). Thus, a safe and effective ZIKV vaccine for pregnant women to prevent in utero ZIKV infection is of utmost importance. Murine models of ZIKV infection are limited by the fact that immunocompetent mice are resistant to ZIKV infection. As such, interferon-deficient mice have been used in some preclinical studies to test the efficacy of ZIKV vaccine candidates against lethal virus challenge. However, interferon-deficient mouse models have limitations in assessing the immunogenicity of vaccines, necessitating the use of immunocompetent mouse pregnancy models. Using the human *stat2* knock-in (hSTAT2KI) mouse pregnancy model, we show that vaccination with a purified formalin-inactivated Zika virus (ZPIV) vaccine prior to pregnancy successfully prevented vertical transmission. In addition, maternal immunity protected offspring against postnatal challenge for up to 28 days. Furthermore, passive transfer of human IgG purified from hyper-immune sera of ZPIV vaccinees prevented maternal and fetal ZIKV infection, providing strong evidence that the neutralizing antibody response may serve as a meaningful correlate of protection.

## Introduction

Zika virus (ZIKV) is a mosquito-borne arbovirus^1,2^ which is associated with teratogenic effects on fetal development when contracted during pregnancy, resulting in microcephaly and other neurological defects termed congenital Zika syndrome (CZS)^3–5^. The WHO declared ZIKV infection as a Public Health Emergency of International Concern in February 2016^6,7^, precipitating the development of a number of preclinical Zika vaccine candidates^8,9^. A few of these vaccine candidates have been shown to be safe and immunogenic in clinical trials^10^. However, the efficacy of these vaccine candidates has not been examined in pregnant women.

Previously, a Zika purified inactivated virus (ZPIV) vaccine candidate was shown to induce durable protective immunity in non-pregnant mice and macaques^11,12^. Additionally, the same vaccine candidate was evaluated in four phase 1 clinical trials, causing only self-limited mild adverse events (e.g., redness and swelling at the injection site, fever, myalgia) and robust immunogenicity in humans has been reported^13–15^. It is critical to demonstrate the efficacy of ZPIV against ZIKV infection during pregnancy. We previously showed that prenatal vaccination of ZPIV prevented ZIKV-induced fetal demise in immunocompetent C57BL/6 mice and induced durable immunity in marmosets that was protective up to 72 weeks post-vaccination against ZIKV challenge during pregnancy^16^. To further demonstrate the efficacy of ZPIV in preventing vertical transmission of ZIKV, here we have examined the effect of ZPIV in human *stat2* knock-in (hSTAT2KI) mice. The NS5 protein of ZIKV binds and degrades STAT2 protein in humans, resulting in the inhibition of type I interferon signaling and enhancing susceptibility to infection^17^. However, ZIKV does not target mouse STAT2, rendering wild-type immunocompetent mice resistant to infection. Human STAT2KI mice, generated by replacing the mouse *stat2* gene with the human *stat2* gene in C57BL/6 mice, become susceptible to ZIKV, similar to humans. It was previously reported^18^ that the mouse-adapted African lineage Dakar strain of ZIKV (ZK-DAK-MA) penetrated the placental barrier and infected the fetus during pregnancy in hSTAT2KI mice. In this study, we show the ability of ZPIV to prevent vertical transmission of ZK-DAK-MA in hSTAT2KI mice. Maternal immunity also protected offspring from ZIKV infection up to 28 days after birth. In addition, we showed that passive antibody transfer of ZPIV-elicited human immune antibodies prevented infection. These results demonstrate that ZPIV is efficacious in preventing vertical transmission of ZIKV and ZIKV-associated pathology in the mouse placenta and fetal brain, and that virus-neutralizing antibodies may act as a correlate of protection. The promising results in this preclinical study encourage further research on ZPIV vaccination during pregnancy.

## Results

### Protection by ZPIV against ZIKV challenge during pregnancy in hSTAT2KI mice

**Fig. 1:**
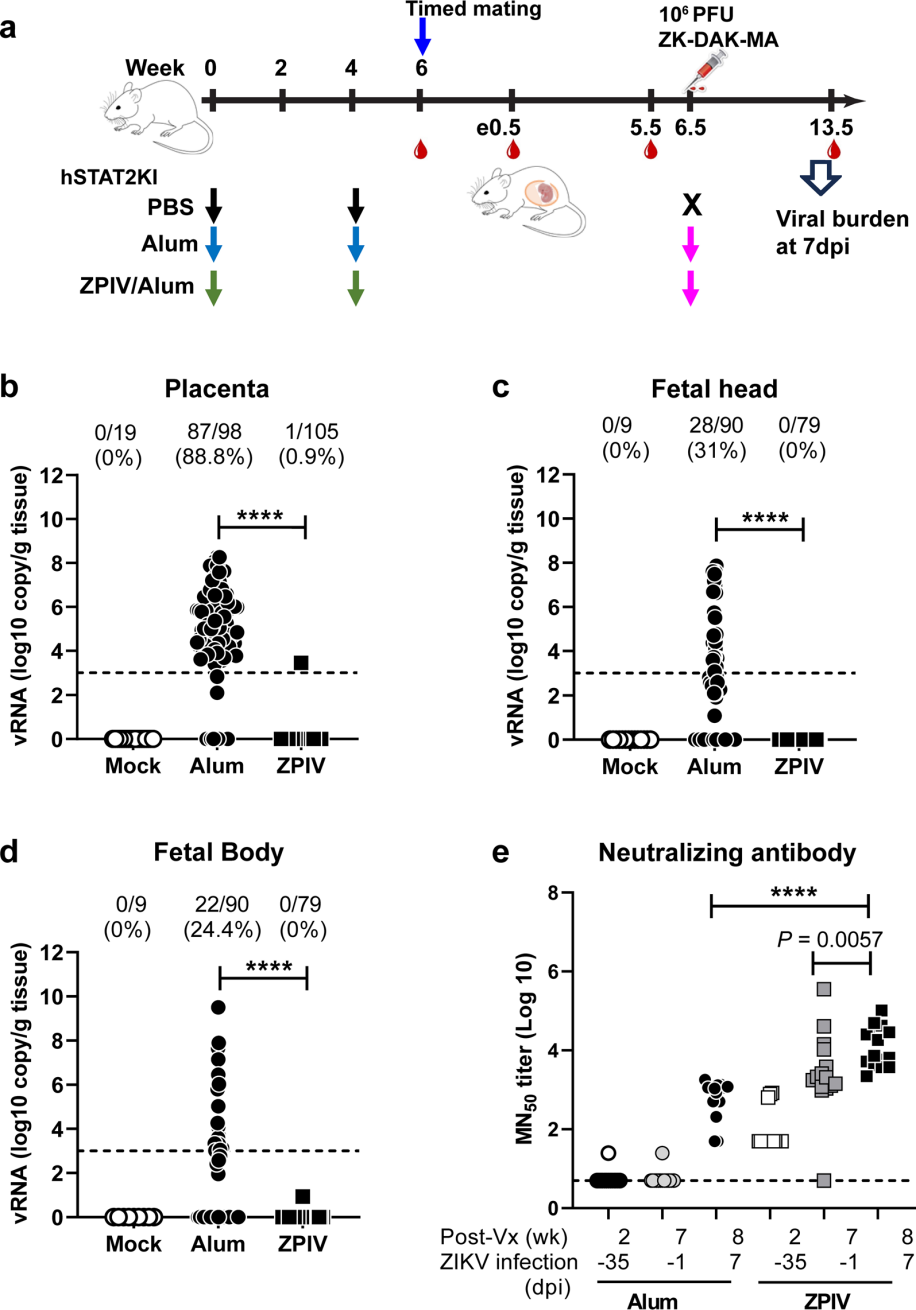
Prevention of vertical transmission by ZPIV in pregnant hSTAT2KI mice. Five-week-old female hSTAT2KI mice were intramuscularly prime-boost immunized with 1 µg alum-adjuvanted ZPIV (square), alum alone (solid circle) or PBS (open circle) at weeks 0 and 4. At week 6, females (11 weeks old) were mated and i.v. injected with 10^6^ PFU ZK-DAR-MA at e6.5. Dams were bled at day -35, -1 (e5.5), and 7 dpi (e13.5), and were sacrificed and examined at 7 dpi (**a**). Not all plug-detected mice were truly pregnant, hence the number of animals examined in each of the experiments varied. Two independent experiments were performed, and data were compiled for each group, mock-control (*n* = 3), alum (*n* = 14), and ZPIV (*n* = 15). Viral RNA levels were determined at 7 dpi in the placenta (**b**), fetal head (**c**), and fetal body (**d**) using RT-qPCR. Virus-neutralizing antibody levels in maternal blood serum samples were assessed using a microneutralization assay, presented as log10 MN_50_ titers (**e**). Individual symbols represent individual samples. Data presents mean (±standard deviation) within the groups. Statistical significance (*P* < 0.05) between the alum and ZPIV groups was determined using the Mann–Whitney test for the analysis of viral RNA levels and the ANOVA test for the analysis of neutralizing antibody titers. ****, *P* < 0.0001. Dashed lines in **b**–**d** indicate the limit of quantitation (Ct value ≤35). Dashed line in **e** indicates the limit of detection, <1:10 dilution.

We first examined the ability of ZPIV to prevent vertical transmission of ZIKV during pregnancy. Prior to pregnancy, female mice were immunized twice with either alum adjuvanted 1 μg ZPIV or alum alone, at a 4-week interval. Mice injected with PBS were included as virus-free controls (mock-control). Two weeks after the boost (second dose), the females were mated. At embryonic day 6.5 (e6.5), mice were challenged with ZIKV and sacrificed at 7 days post-infection (dpi) (Fig. 1a). The number of animals initially assigned to each treatment and the number of animals examined per group were different and vary per group because not all plug-detected mice were truly pregnant, which could be determined at the time of termination at 7 dpi. We conducted two independent studies and presented data which were compiled per group: mock-control (*n* = 3), alum (*n* = 14), or ZPIV (*n* = 15) (Fig. 1). In the alum group, viral RNA (vRNA) was detected in 88.8% (87 out of 98) of the placentas (Fig. 1b). Viral RNA was detected in approximately 31% of the fetal heads and 24% of the fetal bodies of the alum group (Fig. 1c, d). In contrast, in the vaccinated group, only one out of 105 placentas had detectable vRNA, although low in copy number, which is significantly different (*p* < 0.0001) from the alum group. Furthermore, the vRNA was not detected or was below the limit of quantitation in the fetal head and body of the vaccinated group, similar with the mock-control group. At e5.5, 1 day prior ZIKV challenge, we assessed virus-neutralization (VN) titers. While VN titers in the alum group remained at baseline, with a geometric mean log10 MN_50_ titer of 0.7, VN titers in the ZPIV group reached a geometric mean log10 MN_50_ titer of 3.19 (95% confidence interval, 2.5–4.1), exceeding the VN titer of 2.4, which had been determined previously as the threshold of protection^11,16^. At 7 dpi, VN titers in the ZPIV group (geometric mean log10 MN_50_ titer 4.06 with 95% C.I., 3.8–4.3) increased, which were significantly higher than the titers in the alum group (log10 MN_50_ titer 2.7 with 95% C.I 2.4–3.1) (*p* < 0.0001) (Fig. 1e). Additionally, in the ZPIV group, the neutralizing antibody titers at 7 dpi (e13.5) were significantly higher (*p* = 0.0057) than the titers at e5.5, consistent with the stimulation of immune memory upon ZIKV challenge^16^.

### Prevention of ZIKV-associated pathology in the placenta and fetal brain

**Fig. 2:**
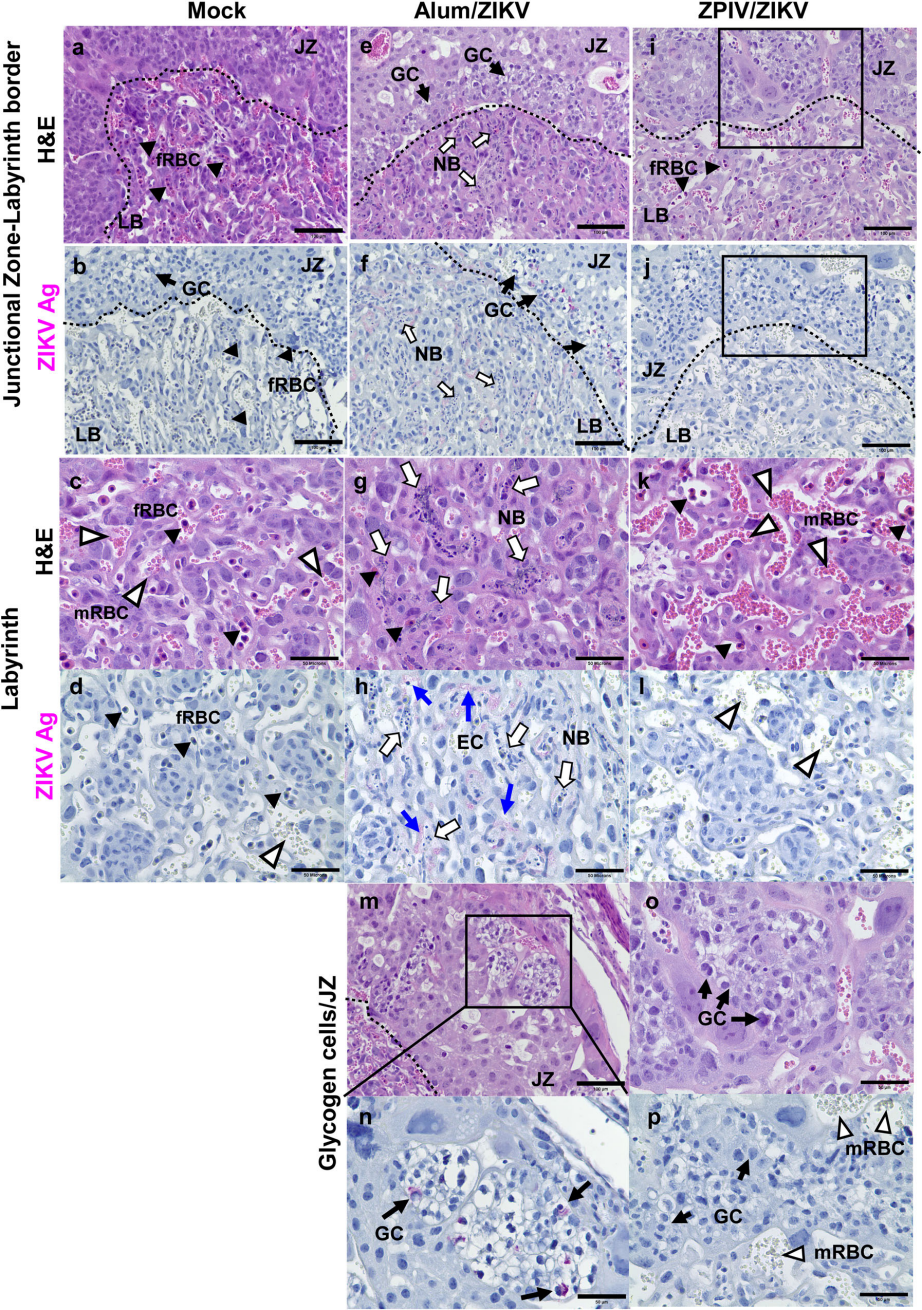
ZPIV vaccination prevents ZIKV-associated pathology in hSTAT2KI mice. Representative images of the placentas of mock-control (**a**–**d**), alum (**e**–**h** and **m**, **n**), or ZPIV (**i**–**l** and **o**, **p**) groups assessed at 7 dpi. Glycogen cells (GC) are lined at the border (dotted line) of the junctional zone (JZ, top) and the labyrinth zone (LB, bottom). In the alum group (**e**–**h**), both the junctional zone (**e**) and the parenchyma of the labyrinth zones (**g**) are expanded, and necrotic bodies (white arrows) are scattered throughout the labyrinth. The loss of fRBC in the labyrinth is evident in the alum group (**e**, **g**). ZIKV envelope (ENV) proteins (pink) are identified by staining with pan-flavivirus mouse monoclonal antibody clone D1-4G2-4-15 of the serial sections (the second and fourth rows) showed virus-infected GC in the JZ (**f**) and in a higher magnification (**n**) of the boxed area (**m**), but not in the ZPIV group (**j**, **p**). In the labyrinth (**f**, **h**), the focal necrosis was found in the vicinity where viral ENV proteins (blue arrows) present mostly in trophoblast cells and fetal endothelial cells (**h**), while viral ENV proteins were absent in the placenta of the ZPIV group (**j**, **l**, **p**). Maternal red blood cells, mRBC; fetal red blood cells, fRBC; necrotic body, NB; endothelial cells, EC. Scale bars in **a**–**l** indicate 100 μm, and scale bars in **m**–**p** indicate 50 μm.

At 7 dpi, histopathology of the placentas from mock-control (PBS), alum, and ZPIV groups was examined (Fig. 2). Compared with the placenta of the mock-control group (Fig. 2a–d), inflammation was evident in the alum group (Fig. 2e–h), with a thickened junctional zone and parenchyma of the labyrinth zone in the placenta (Fig. 2e, g). Additionally, it was noted that nucleated fetal red blood cells were scarce and necrotic bodies were scattered throughout the labyrinth zone of the placenta in the alum group when compared with the mock-control (Fig. 2a, c) and ZPIV groups (Fig. 2i, k). Some focal necroses were observed in the proximity of viral envelope (ENV) proteins, which were present mostly in endothelial cells and trophoblasts throughout the labyrinth in the alum group (Fig. 2f, h), while no viral protein was detected in the ZPIV group (Fig. 2j, l). Furthermore, viral ENV proteins were also present primarily in glycogen cells, characterized by a vacuolated glycogen-rich cytoplasm at the border of the junction-labyrinth zone in the alum group (Fig. 2f, m, n), which were absent in the ZPIV group (Fig. 2j, o, p). These results show that ZPIV successfully prevents viral infection and virus-induced inflammation in the placenta.

**Fig. 3:**
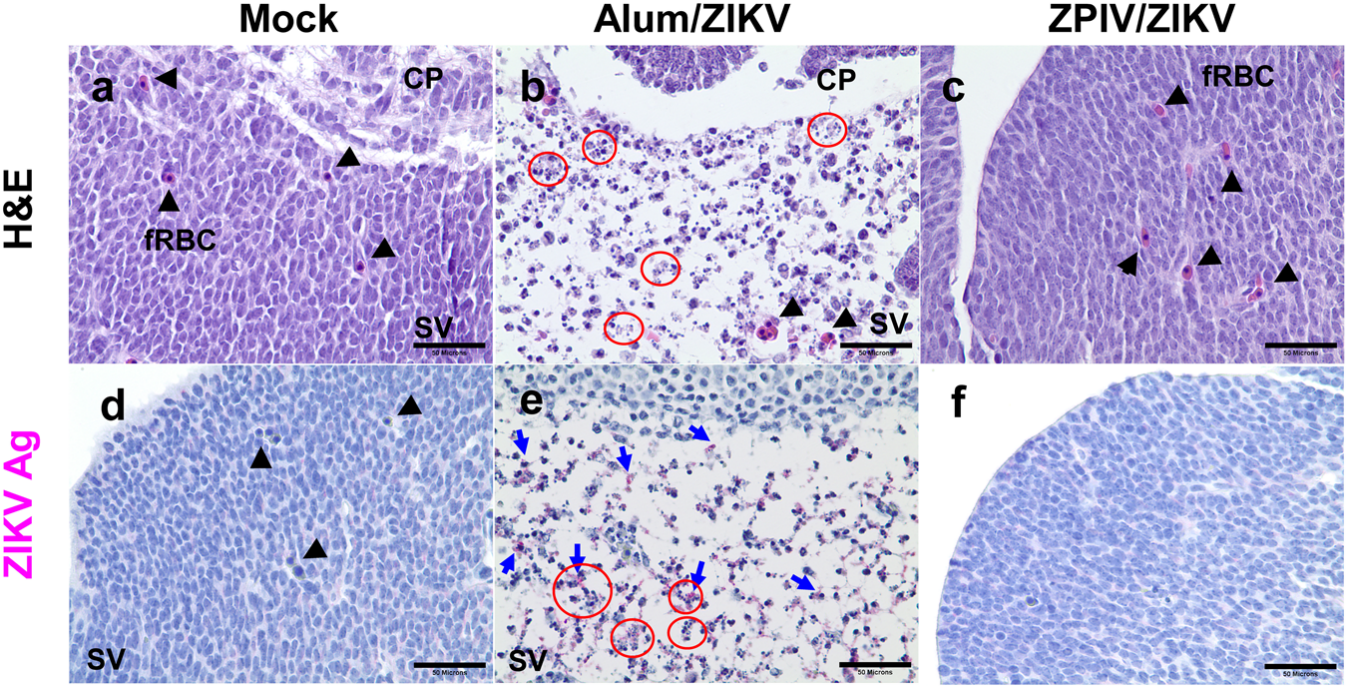
Prevention of brain pathology by ZPIV vaccination in hSTAT2KI mice. Representative images of the fetal brain from the mock-control (**a**, **d**), alum (**b**, **e**), or ZPIV (**c**, **f**) groups at 7 dpi. In the alum group, necrotic bodies (red circle) are scattered throughout the subventricular zone (SV), evident with loss of cellular density (**b**), which are absent in the fetal brain from the ZPIV group (**c**) comparable with those from the mock group (**a**). Coinciding with the brain pathology, ZIKV ENV proteins (blue arrow) were present in the subventricular zone in the alum group (**e**), but not in the ZPIV group (**f**), similar with the mock group (**d**). CP, choroid plexus, fRBC, fetal red blood cells, (arrowhead); SV, subventricular zone. Scale bars = 50 μm.

Analysis of fetal brain tissue showed that when compared with the mock-control (Fig. 3a), the fetal brain from the alum group presented focal necrosis, resulting in loss of cellular density in the subventricular zone (Fig. 3b) at 7 dpi. In contrast, the brains of the ZPIV-vaccinated group (Fig. 3c) showed no signs of inflammation and pathology. As expected, viral ENV proteins were absent in the brains of the mock-control group (Fig. 3d). However, in the alum group, they were scattered in the subventricular zone, where extensive necrosis was found in the fetal brain (Fig. 3e). The fetal brains from the ZPIV group were free of viral ENV proteins and inflammation (Fig. 3f). Taken together, histopathological analyses of the placenta and the fetal brain strongly support the protective efficacy of ZPIV against prenatal ZIKV challenge.

### Maternal immunity protects against postnatal challenge

**Fig. 4:**
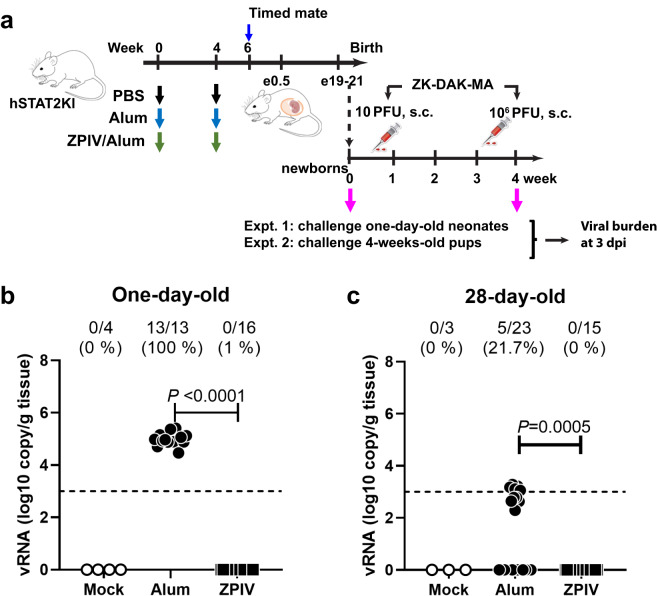
Protection of offspring by maternal immunity against postnatal ZIKV challenge. Female mice were intramuscularly injected with PBS (mock), alum, or 1 μg alum-adjuvanted ZPIV at weeks 0 and 4. At week 6, the mice were mated and allowed to complete term (**a**). One-day-old neonates born from mock (*n* = 4), alum (*n* = 13), or ZPIV (*n* = 16) dams were subcutaneously injected with 10 PFU ZK-Dak-MA (**b**). Twenty-eight-day-old pups (C) born from mock (*n* = 3), alum (*n* = 15), or ZPIV (*n* = 23) dams were subcutaneously injected with 10^6^ PFU of the virus. At 3 dpi, fetal heads (**b**) or brains (**c**) were examined to determine the level of viral RNA using RT-qPCR. Individual symbols represent individual samples. Data presents mean (±standard deviation) within the groups. The Mann–Whitney test was used to determine significant differences in viral RNA levels between groups. The dotted line indicates the limit of quantitation (Ct value ≤35).

Maternal antibodies which cross the placenta during pregnancy or are found in the colostrum during the breast-feeding period play an important role in the protection of newborns^19,20^. In this study, we examined whether ZPIV-elicited maternal antibodies protect offspring born from vaccinated dams against postnatal ZIKV challenge. To test the role of maternal antibodies transferred across the placenta and through breast milk, we chose to infect one-day-old neonates and 28-day-old pups, which is one week after weaning, respectively. Five dams of each of the mock-control, alum, and ZPIV-immunized groups were allowed to complete term without ZIKV challenge during the pregnancy. Offspring were challenged with 10 PFU or 10^6^ PFU of ZIKV-DAK-MA at 1 day old and 28 days old, respectively, based on the dose study (Supplementary Fig. 1) and examined at 3 dpi according to previous reports^21,22^ (Fig. 4a). As expected, no viral RNA was detected in the mock-control group. Viral RNA was detected in 100% of the neonatal heads from the alum group whereas none was detected in heads from the ZPIV group during the suckling period (Fig. 4b). In addition, vRNA was not detected in the brains of the 28-day-old juvenile pups in the ZPIV group, whereas the alum group showed detectable vRNA at relatively low levels (Fig. 4c). This suggests that 28-day-old mice were gaining resistance to viral infection, possibly due to developing immunity. These data indicate that ZPIV-elicited maternal immunity provides protection to offspring against ZIKV infection at one or 28 days after birth, although, in the absence of extensive kinetics, the data cannot distinguish whether maternal antibodies delay or prevent infection.

### Protection by ZPIV vaccination-derived human polyclonal IgG transfer against prenatal ZIKV challenge in hSTAT2KI mice

**Fig. 5:**
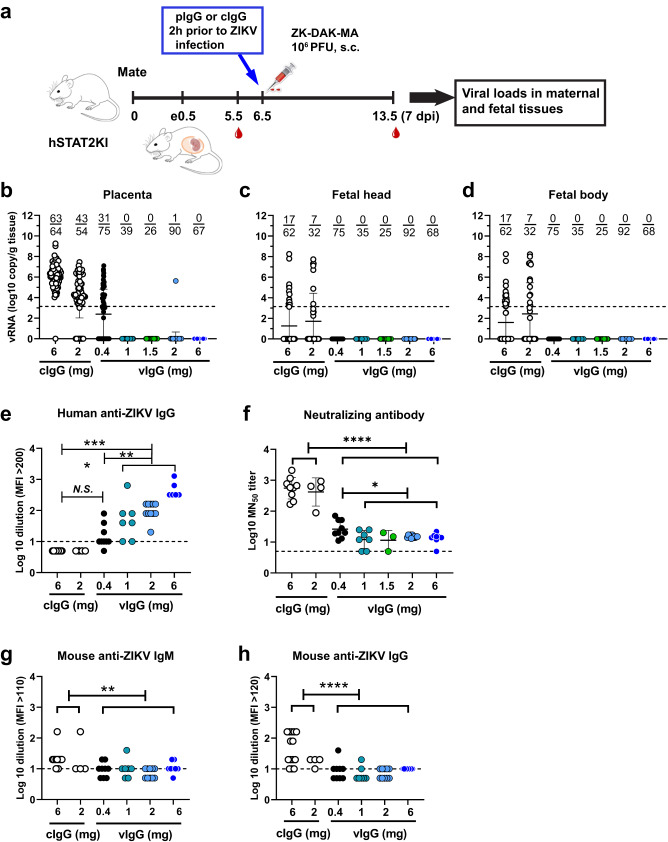
Passive antibody transfer prevents prenatal ZIKV infection. At e6.5, 2 h prior to ZIKV infection, naïve dams were i.v. injected with the indicated doses of purified IgG prepared from the vaccine-derived immunoglobulin G (vIgG) or unvaccinated control IgG (cIgG), followed by ZIKV challenge. At 7 dpi (e13.5), the dams were sacrificed (**a**), and viral RNA (vRNA) was detected in the placenta (**b**), fetal head (**c**), and fetal body (**d**) using RT-qPCR. Compiled data were from two independent studies examining different dose ranges: 6 mg (*n* = 8), 2 mg (*n* = 11), 1.5 mg (*n* = 3), 1 mg (*n* = 5), 0.4 mg (*n* = 9) vIgG per dose. Control IgG at the highest dose was included as a negative control in each study (6 mg, *n* = 7; 2 mg, *n* = 5). The numerators indicate the number of samples positive for vRNA over the number of samples examined per group. Dashed lines (**b**–**d**) indicate the limit of quantitation. One-way ANOVA comparison between the control group combined between 6 mg and 2 mg control IgG (*n* = 118 per the placenta and *n* = 94 per the fetal head and body) and individual treatment groups showed significant differences (*p* < 0.0001) in viral RNA levels in the placenta, fetal head, and fetal body. At 7 dpi, the donor, vIgG titers were detected (**e**) and virus-neutralizing antibody titers were examined using a microneutralization assay (**f**). Mouse IgM (**g**) and IgG (**h**) specific for ZIKV in the recipients were detected using a Luminex assay. Dashed lines (**e**–**h**) indicate the limit of detection. Antibody titer was determined as the highest serum dilution of MFI values above average MFI (+2 S.D.) of naïve serum control. Log-transformed data were compared between the cIgG group and each of the vIgG groups. N.S., *p* > 0.05; *p < 0.05; **p < 0.001; ****p < 0.0001.

To test whether neutralizing antibodies are potential correlates of protection against ZIKV, we conducted a passive transfer study into naïve pregnant mice before the ZIKV challenge. Immune serum samples from healthy unvaccinated humans and humans after hyper-immunization with ZPIV^15^ were pooled, purified, and characterized. The neutralizing activity (MN_50_ titer) of the purified IgG stock (47.7 mg/ml) was 16,674 (Supplementary Table 1 and Supplementary Fig. 2). De-escalating doses of the vaccine-derived human purified IgG (vIgG) were intravenously injected into naïve dams 2 h prior to ZIKV infection at e6.5, as outlined in Fig. 5a. Mice pre-treated with a dose equal to and higher than 1 mg (eq. 37 mg/kg) vIgG were completely protected against perinatal challenge, indicated by the absence of vRNA in the fetal head or body, and in the placenta of all but 1 of the group treated with 2 mg vIgG. However, 31 of 75 placentas (41.3%) from the group that received 0.4 mg (eq.15 mg/kg), showed detectable vRNA (Fig. 5b). Despite placental infection in the 0.4 mg group, all doses of vIgG successfully prevented the cross-placental transmission of the virus to the fetus, as indicated by the absence of vRNA in the fetal brain and body (Fig. 5c, d).

### Antibody response of the recipients

At 7 dpi (e13.5), we detected virus-specific human donor IgG, in a dose-dependent manner, in serum samples of the recipients (Fig. 5e). The log-transformed geometric mean (1.2) of detectable donor IgG of the group which received 0.4 mg was not significantly different from the control IgG group but was significantly lower (*P* < 0.0001) than the levels (a range of 1.7–2.6) of the groups which received 1 mg and higher doses. In contrast, virus-neutralizing antibody titers (log10 MN_50_ titers) in the control IgG groups (geometric means of 2.6–2.7, *n* = 13), likely the primary antibody response of the recipients, were significantly higher (*P* < 0.05) than those of all groups that received vIgG. Among recipients of different doses of vIgG, the lower neutralizing activities (geometric means of 1.0–1.18) detected across the 1–6 mg vIgG doses compared with the 0.4 mg group (geometric mean, 1.39) likely reflect a weaker primary antibody response of the recipients because of more complete neutralization of virus by the passive transfer of vIgG (Fig. 5f).

To test this possibility, we examined the maternal serum samples for host (mouse) anti-ZIKV antibody response. There was little discernible difference in virus-specific IgM levels between the mice that received vaccine-derived antibodies, or not (Fig. 5g). However, virus-specific IgG levels were higher in the mice receiving control antibodies compared with those receiving all doses of vIgG (Fig. 5h), indicating that the neutralizing antibodies detected in Fig. 5f reflected the host response.

## Discussion

Safe and effective vaccines are urgently needed for preventing ZIKV infection in pregnant mothers and fetuses. In the current study, using an immunocompetent mouse pregnancy model of ZIKV infection, we have shown that ZPIV vaccination prior to pregnancy successfully prevented ZIKV transmission from mother to fetus in utero as assessed by vRNA analysis and histological analysis of placental and brain tissue. Moreover, the vaccine-induced maternal immunity, presumably transferred in utero or during suckling, provided protection to pups against postnatal challenge. Finally, the passive transfer of highly neutralizing human antibody conferred protection in a dose-dependent manner, suggesting that neutralizing antibodies play an important role in the control of ZIKV infection and preventing vertical transmission, which is supported by the little or no primary antibody response in the recipients. The current results in hSTAT2KI mice strongly support the efficacy of ZPIV in protecting both pregnant mothers and fetuses against ZIKV infection during pregnancy and highlight the potential of ZPIV as a potent vaccine candidate for pregnant women in future Zika outbreaks. These data also reinforce the rationale for developing antibody-based treatments for ZIKV infection during pregnancy.

Although ZIKV fails to establish productive transplacental replication in immunocompetent mice unless it is injected via the intracranial^23^ or the intra vaginal^24^ route, the virus causes placental insufficiency resulting in fetal demise^22,25–27^. In contrast, similar to humans, ZIKV is transmitted from mother to fetus and replicates in the fetus of hSTAT2KI mice^18^, making hSTAT2KI mice a relevant preclinical model. At 7 dpi, viral ENV proteins are predominantly on the fetal side of the placenta in the alum group. ZIKV infects not only endothelial cells and trophoblasts in the labyrinth, but also glycogen cells in the junctional zone. These cells are critical to the survival of the fetus^28^ because they serve as the energy source for placental and fetal development during pregnancy^29^. We speculate that the infection of glycogen cells with the virus leads to a shortage of the nutrient supply required for placental development, consequently contributing to placental insufficiency and fetal growth restriction. Furthermore, the alum group showed evidence of neurotropic destruction in the fetal brain, in agreement with the previous reports^30,31^. In contrast, the prime-boost vaccination successfully prevented vertical transmission and Zika-associated pathology in the placenta and fetal brain in hSTAT2KI mice.

Maternal immunity plays an important role in the protection of fetuses and infants against infectious pathogens^20,32,33^. In humans, IgG transfer across the placenta begins in the late first trimester (~17 weeks of gestation) and increases towards term^34,35^. To examine the efficacy of maternal antibodies, we infected 1-day-old neonates to assess the protection by antibodies transferred across the placenta. Infection of 28-day-old pups allowed assessment of protection by the antibodies obtained from breast milk after suckling. In agreement with previous studies using immunocompetent Balb/C^11^ and C57BL/6^16^ as well as in IFN-deficient mouse models^21,36^, the current study showed that the suckling neonates and juvenile pups born from the ZPIV-vaccinated hSTAT2KI mouse dams were protected against postnatal challenge. One caveat of the current study is that in the absence of thorough kinetics, we can’t distinguish whether the maternal antibody was sufficient to prevent infection or delay infection. Regardless, the protective effect of passively transferred purified antibody demonstrated that neutralizing antibodies play a crucial role in controlling the virus and provided complete prevention of vertical transmission in hSTAT2KI mice. An interesting observation is that the recipients which were administered doses of 1 mg and higher showed relatively high levels of donor antibodies, but low neutralizing activity. This contrasts with the recipients who received 0.4 mg, in which protection was incomplete. The dose effect in these studies underlines the importance of optimization of the dose and frequency of antibody-based therapy for efficacy in humans. The current results clearly demonstrate that virus-neutralizing antibody confers protection from ZIKV infection in the absence of memory B cells.

Despite the significant findings, there are limitations of the current studies. The current studies do not directly address the role of memory B cells in protective immunity. We also didn’t investigate vaccine-induced T-cell responses, although it has previously been shown that ZPIV elicits a virus-specific T-cell response in non-pregnant cynomolgus macaques^12^, indicating that T-cell responses may contribute to vaccine-induced protection. Rather, we focused on the prophylactic effect of virus-neutralizing antibodies. Although the current mouse studies establish proof of concept, it is critical to determine whether the results can be translated to humans. Further research into the therapeutic potential of antibody-based treatments is warranted and could establish the validity of virus-neutralizing antibodies as a possible treatment for pregnant women in the next ZIKV outbreak.

In conclusion, the current study showed that ZPIV vaccination prior to pregnancy prevented transplacental transmission in pregnant immunocompetent hSTAT2KI mice. In addition, the vaccine elicited maternal immunity which protected offspring from postnatal challenge. Finally, the passive transfer of purified human polyclonal antibodies from hyper-immune vaccinees protected the pregnant mice, showing that antibodies are an important correlate of protection. These results further validate the use of hSTAT2KI mice as a preclinical model and indicate important areas for further investigation for developing clinical therapeutics using the ZPIV vaccine.

## Methods

### Ethics statement

The research was conducted in compliance with the Animal Welfare Act and other federal statutes and regulations relating to animals and experiments involving animals and adheres to principles stated in the Guide for the Care and Use of Laboratory Animals, NRC Publication, 1996 edition. All mouse studies were conducted at Trudeau Institute following the protocol, which was approved by the Institutional Animal Care and Use Committee (IACUC) in accordance with the Guide for Care and Use of Laboratory Animals of the National Institutes of Health.

Serum samples from human vaccinees were from the RV478 study, “A Phase 1, First-in-human, Double-blinded, Randomized, Placebo-controlled Trial of a Zika Virus Purified Inactivated Vaccine (ZPIV) With alum adjuvant in Healthy Flavivirus-naive and Flavivirus-Primed Subjects”. The WRAIR Institutional Review Board (IRB) approved the protocol prior to the study initiation. Written informed consent was obtained from all participants before screening. The investigators have adhered to the policies for the protection of human participants as prescribed in AR 70–25. The trial is registered at ClinicalTrial.gov number: NCT02963909.

### Experimental design

Breeding pairs of humanized STAT2 knock-in mouse (C57BL/6-*Stat2*^*tm1.1(STAT2)Diam*^/AgsaJ) were purchased from the Jackson Laboratory (Bar Harbor, MA) and bred and maintained in the Association for Assessment and Accreditation of Laboratory Animal Care International accredited animal facility of Trudeau Institute. Adult (5–6 weeks old) female hSTAT2KI mice were intramuscularly prime-boost immunized with 1 µg alum-adjuvanted ZPIV or alum alone (*n* = 10/group) at week 0 and 4 (Fig. 1a), aiming to examine 8 pregnant dams per group. Two weeks after the boost dose, the female mice (11–12 weeks old) were co-housed with males in a 3:1 ratio and checked daily for the detection of copulatory plugs (e0.5), as described previously^22^. Firstly, to examine the efficacy of vaccination, at embryonic day 6.5 (e6.5), female mice were anesthetized with isoflurane and injected with 100 μL of 10^6^ PFU of ZIKV-DAK-MA via the retro-orbital sinus vein and euthanized 7 days after infection (e13.5) by CO_2_ inhalation. Five mock-control animals with PBS injection were included per experiment to serve as virus-free controls. Maternal blood was collected at 0, 2, 4, 6, and 7 (e6.5) weeks via the mandibular vein prior to ZIKV infection. Not all plug-detected mice were truly pregnant, hence the number of animals examined in each of the experiments varied. Accordingly, the number of animals examined per experiment is indicated in each figure legend. Secondly, to examine the protection of offspring from the postnatal challenge, 5 dams per group were allowed to complete pregnancy without ZIKV challenge during the pregnancy, and the offspring were challenged with 10^1^ or 10^6^ PFU of ZIKV at one-day (*n* = 13–16 /group) or 28-days after birth (*n* = 15–23/group), respectively. We tested ranges of viral dose in unvaccinated neonates to determine the experimental dose used in these experiments (Supplementary Fig. 1). Then, the pups were euthanized at 3 dpi for examination as described previously^21^ (Fig. 4a). Some pups were lost due to cannibalism after birth. Only the live pups at the end of the study protocol were examined. Finally, to examine whether virus-neutralizing antibodies are correlates of protection, naïve pregnant dams were injected with de-escalating doses (6–0.4 mg) of purified human IgG from ZPIV-vaccinated individuals (3–11 dams/dose) or 2 mg or 6 mg control human IgG (5–7 dams/dose) prior to ZIKV infection at e6.5 (Fig. 5a). Two hours after passive transfer of the antibody, dams were challenged with 10^6^ PFU of ZIKV-DAK-MA. Two independent studies were performed to examine different dose ranges; one study examined 6, 2, and 0.4 mg per dose and the other study examined 2, 1.5, 1, and 0.4 mg per dose. As controls, the highest doses of control human IgG per study (6 and 2 mg, respectively) were included.

At 7 dpi, mice were euthanized by CO_2_ overdose, terminally bled, and maternal spleens, lymph nodes, and uterus were removed, and fetuses and placentas were separated using an aseptic technique. Randomly selected fetuses, 2–3 per dam, were placed in tissue cassettes and immersed in 10% neutral buffered formalin (NBF, Fisher Scientific) for histology. The rest of the fetuses were decapitated, and fetal heads and bodies were frozen immediately in liquid nitrogen and kept at −80 °C until further processing for RNA isolation.

### Infection dose selection for neonate and juvenile pups

Naïve one-day-old neonates (*n* = 5) were subcutaneously injected with a dose of 1, 10, or 100 PFU of ZIKV-DAK-MA and examined for viral burden in the brains and spleens at 3 dpi according to the previous report^18^^,^^21^. Some neonates were found dead, and it was not clear whether the deaths were due to lethal infection with the virus or cannibalism as there was no remaining corpse. Twenty-eight days old juvenile pups (*n* = 5) were injected with 10^3^ or 10^4^ PFU of ZIKV -DAK-MA by footpad route and examined the brains at 3 dpi. Based on these results, we selected the infection doses for the postnatal challenge study shown in Fig. 4.

### Viruses, cells, and titration

The mouse -adapted African lineage Dakar strain of ZIKV (ZK-DAR-MA) was a generous gift from Dr. Michael Diamond (Washington Univ., St. Louis) and the virus was propagated in Vero cells (CCL-81, ATCC), as described previously^18^. Viral titers of the stock were determined by plaque and focus-forming assays on Vero cells, as described previously^22^.

### Zika purified inactivated virus (ZPIV) vaccine

ZPIV vaccine was developed, prepared, and provided by the Walter Reed Army Institute of Research (WRAIR)^11^. ZPIV contains a chromatographic-column-purified, formalin-inactivated Puerto Rico strain of Zika virus, PRVABC59, that was initially obtained from the Centers for Disease Control and Prevention (Fort Collins, CO, USA) and cultured and passaged in a qualified Vero cell line. After purification and inactivation, the virus was absorbed in a 1:1 ratio with 1 mg/mL alum (Alhydrogel, Brentagg Biosector, Frederikssund, Denmark). Alum-absorbed ZPIV was prepared at the concentration of 10 μg/mL.

### Purification of human polyclonal immunoglobulin G from vaccinees and normal donors

Serum samples from flavivirus naïve individuals having received three ZPIV immunizations were selected based on high ZIKV neutralizing antibody geometric mean titers^15^. Samples from two study visit days (252 and 308), one- and three-months post third ZPIV dose, respectively, from 19 individual donors, were heat-inactivated, centrifuged at 20,000 × *g* for 5 min and pooled. Normal human serum was obtained commercially (Sigma, H4522) and was confirmed negative for ZIKV neutralization. All materials were diluted 10:1 with 10X PBS pH 7.4 and loaded on custom Protein G Sepharose (Cytiva, 17061801) columns by recirculating the flow-through up to four times. After extensive washes in 1X PBS pH 7.4, bound IgG were eluted with 0.5 M Acetic Acid, pH 3.0, quickly neutralized with 3 M Tris, concentrated and buffer exchanged to 1X PBS pH 7.4 and sterile filtered. Quantitation of material was performed on a Nanodrop spectrophotometer using IgG setting. Purity was assessed by SDS-PAGE and identity was confirmed by western blot (Supplementary Figure 2). Endotoxin levels were measured using a LAL assay (Lonza). Purified materials were functionally tested for ZIKV neutralization (Supplementary Table 1).

### RNA isolation

Frozen tissues were treated with RLT buffer (Qiagen) containing β-mercaptoethanol (β-ME, Sigma-Adrich) at a concentration of 100 mg/mL and homogenized with stainless steel beads using a TissueLyzer II instrument (Qiagen). For the lipid-rich brains, Trizol (Thermo-Fisher) was added to prepare homogenates followed by the addition of chloroform. RNA extractions from the aqueous phase were carried out using the RNeasy mini kit (Qiagen) according to the manufacturer’s instructions^22^. RNA pellets were resuspended in 60 µL of RNase-free distilled water, quantified using a NanoDrop 2000 (NanoDrop Technologies, Wilmington, DE), and stored frozen at −70 °C.

### One-step real-time quantitative reverse transcription-polymerase chain reaction (qRT-PCR)

For ZIKV RNA detection, one-step qRT-PCR was performed on a 7500 Fast Real-time PCR System (Applied Biosystems). As described previously^37^, ZIKV-specific primers and probe sequences are: forward, 5′-CCGCTGCCCAACACAAG-3′, reverse 5′-CCACTAACGTTCTTTTGCAGACAT-3′, probe 5′-/56-FAM/AGCCTACCT/ZEN/TGACAAGCAGTCAGACACTCAA/3IABkFQ/-3′ (Integrated DNA Technologies). The PCR conditions were optimized using 1 µg total RNA in a 20 µL reaction cocktail containing TaqMan Fast Virus 1-step Master Mix (Applied Biosystems), 5 pM primers, and 20 pM probes (IDT, Coralville, IA). The PCR was performed using the cycling condition of 50 °C for 15 min, 95 °C for 2 min, followed by 45 cycles of 95 °C for 15 s, 60 °C for 30 s, and the data were analyzed with 7500 Fast software (version 1.4). Viral RNA levels were interpolated against standard curves prepared by diluting RNA from uninfected tissue spiked with a known copy number of ZIKV genomic RNA (NR-50244) obtained from BEI Resources (Manassas, VA). As described previously^16^, the limit of detection was defined as the cycle of threshold (Ct) equal to 37 and the limit of quantitation was defined as Ct value ≤35 with 100% positivity of PCR runs of the standard control.

### Microneutralization (MN_50_) assay

Aliquots of the individual serum samples were shipped to WRAIR for examination of virus-neutralizing activity. ZIKV neutralizing antibody titers were determined using a high throughput microneutralizing antibody assay at WRAIR, as described previously^16^. Briefly, all serum samples were heat inactivated at 56 °C for 30 min and diluted in PBS at 1:10 and 8 serial dilutions per sample were tested. The serum dilutions were mixed with 100 PFU of ZIKV PRVABC59 per well. Following incubation at 35 °C for 2 h, the mixtures were added to 96-well plates containing Vero cell monolayers in triplicate wells and the plates were incubated for 4 days. Then, following the washing, fixing, and blocking steps, the plates were incubated with pan-flavivirus monoclonal antibody, clone 6B6-C1 (a gift from J. T. Roehrig, US Centers for Disease Control and Prevention) conjugated with HRP for 2 h. The plates were then washed and incubated with TMB substrate for 50 min at RT. The enzymatic reaction was stopped by adding 1:25 phosphoric acid, and the absorbance was measured - optical density (OD) at 450 nm. Fifty percent microneutralization (MN_50_) titers were determined as the reciprocal serum dilution corresponding to the wells reducing OD values by 50% when compared with wells containing 100 PFU of virus alone.

### Coupling of microsphere beads with the ENV protein of ZIKV

The His-tagged envelope protein of ZIKV was purchased from Sino Biological (Wayne, PA) and coupled to MagPlex beads (Luminex Corp) following the protocol previously described^38^ with minor modifications. Briefly, 6 × 10^6^ microsphere beads were added to an amber tube avoiding photo bleaching, washed in molecular-graded water, and resuspended in 100 µL of 100 mM sodium phosphate monobasic buffer (pH 6.3, Sigma-Adrich). Then, 50 µL of 50 mg/mL sulfo-*N*-hydroxysulfosuccinimide (Thermo Scientific Pierce) prepared in sodium hydrogen phosphate solution (pH 6.3, Sigma-Aldrich) and 50 µL of 50 mg/mL μg of ethyl-3-(3-dimethylaminopropyl) carbodiimide (Thermo Scientific Pierce) were added. The reaction tube was incubated for 20 min at room temperature (RT) on an orbital shaker at 300 rpm. Beads washed with Dulbecco’s phosphate-buffered saline (DPBS) three times were resuspended at a concentration of 10 µg protein/10^6^ beads in a reaction volume of 500 µL. After a brief vortex mix, the tube was incubated for 2 h shaking at 300 rpm at room temperature. Then, the beads were washed with DPBS three times and resuspended in DPBS containing 1% probumin (Sigma-Aldrich) and 0.05% sodium azide (Sigma-Aldrich), counted and adjusted to a desired concentration, and stored at 4 °C until use.

### Luminex assay to detect virus-specific immunoglobulins

Serum samples were incubated at 56 °C for 15 min and prepared at 1:10 or 1:20 dilution in 1x PBS, and then diluted using the twofold serial dilution method. The serum dilutions were added into ProcartaPlex 96-well flat bottom plate (Invitrogen) in duplicate. One hundred microliters of microsphere beads (2.5 × 10^4^ beads/mL, region 43) conjugated with the ENV protein of ZIKV were added per well and plates were incubated for 2 h at RT on an orbital shaker at 300 rpm. Then the plates were placed on a magnetic field plate holder. After incubation for 3 min, the supernatant was dumped and 200 µL1x PBS containing 0.05% Tween 20 (PBST) was added. The washing step was repeated four times. Then, beads were resuspended in phycoerythrine (PE)-conjugated secondary antibody specific for mouse IgG (H + L) (Southern Biotechnology (SBT) 1031-09), or mouse IgM (BD Bioscience 553521), diluted in PBST and incubated for 1 h at RT on an orbital shaker at 300 rpm. The plates were washed four times with PBST. After the final wash, beads were resuspended in 150 μL PBST and plates were shaken for 3 min on an orbital shaker prior to acquisition on a MAGPIX xMAP reader (Luminex) using the xPONENT software (version 4.1).

Pan-flavivirus mouse monoclonal antibody clone 4G2 (D1-4G2-4-15, Absolute Antibody) was used as a positive control and naïve mouse serum samples were used as negative controls. In addition, internal controls of the assay per individual plate, quality control 1, MFI 4000, quality control 2, MFI 500 and bead only (MFI <50) were added to monitor the performance of individual assay plates.

### Histology and immunohistochemistry

Tissues were fixed with 10% neutral buffered formalin (Fisher Scientific) and processed into paraffin blocks. Sections were cut at 5 μm thickness and mounted on charged glass slides (Fisher Scientific). One set of slides was stained with hematoxylin and eosin following a previously described protocol^16^. Immunohistochemistry was performed on a second set of slides to stain the ZIKV ENV proteins. Briefly, slides were deparaffinized and rehydrated to distilled water. Antigen retrieval was done in citrate buffer pH 6.0 (Fisher Scientific) for 30 min in a steamer. Endogenous enzyme activity was blocked with BLOXALL (Vector Labs) for 15 min at room temperature (RT). The tissue was then incubated in 2.5% horse serum for 30 min followed by the addition of the primary antibody, rabbit anti-ZIKV envelope IgG (GeneTex) at 1:3000 dilution in 2.5% horse serum. The primary antibody was incubated for 2 h at RT. Slides were washed in PBS followed by incubation with a horse anti-rabbit polymer detection kit, alkaline phosphatase (Vector Laboratories). Slides were washed in PBS and developed using Vector-Red Alkaline Phosphatase substrate (Vector Laboratories). Uninfected tissue was used as a negative control and tissue known to be infected was used as positive controls. Slides were imaged using a Nikon Eclipse Ci microscope and Nikon SPOT 2 digital camera.

### Statistical analysis

All data were analyzed using GraphPad Prism software v 9.2.0 (San Diego, CA). Viral RNA levels were analyzed using unpaired non-parametric Mann–Whitney test between groups. Log-transformed antibody titers were compared using the ANOVA test between groups at indicated time points.

### Reporting summary

Further information on research design is available in the Nature Research Reporting Summary linked to this article.

### Supplementary information

Additional Information

Dataset1

Dataset 2

Dataset 3

REPORTING SUMMARY

## Data Availability

The data that support the findings of this study are available in supplementary information and from the corresponding author upon written request and with permission of WRAIR.
