# Supplementary material for: Zika purified inactivated virus (ZPIV) vaccine reduced vertical transmission in pregnant immunocompetent mice
Source: NPJ Vaccines. 2024 Feb 15;9:32. doi: 10.1038/s41541-024-00823-1 (PMC10869681; doi:10.1038/s41541-024-00823-1)
Supplement: Supplementary file 1 — Additional Information [file 41541_2024_823_MOESM1_ESM.docx]

**Zika purified inactivated virus (ZPIV) reduced vertical transmission in pregnant immunocompetent mice**

In-Jeong Kim^1*^, Michael P. Tighe^1^, Paula A. Lanthier^1^, Madeline J. Clark^1^, Rafael A. De La Barrera^2^, Vincent Dussupt^3, 4, 5^, Letzibeth Mendez-Rivera^3,4,5^, Shelly Krebs^3, 4, 5^, Kelsey L. Travis^1^, Timothy C. Low-Beer^1^, Tres S. Cookenham^1^, Kathleen G. Lanzer^1^, Derek T. Bernacki^1^, Frank M. Szaba^1^, Amanda A. Schneck^1^, Jerrold Ward^6^, Stephen J. Thomas^7^, Kayvon Modjarrad^3^^†^, and Marcia A. Blackman^1^

**Supplementary Information**

**Table of Contents**

Supplementary Figure 1

Supplementary Figure 2

Supplementary Table 1

Supplementary Figure 1. Infection dose selection for one-day-old neonates and 28-day-old mice

Virus-free one-day-old neonates (n=5) were injected subcutaneously with 1, 10, or 100 PFU of mouse adapted ZIKV-DAK and at 3dpi, viral RNA was detected in the head (a) and spleens (b). Twenty-eight days old juvenile mice (n=5) were injected with either 10^3^ or 10^4^ PFU of ZIKV by foot pad. At the indicated time after infection, viral RNA was detected in the spleens (n=5 per group, c). Some neonates infected with 1 PFU were lost due to cannibalism. Symbols indicate individual samples. Dotted lines indicate limit of quantitation.


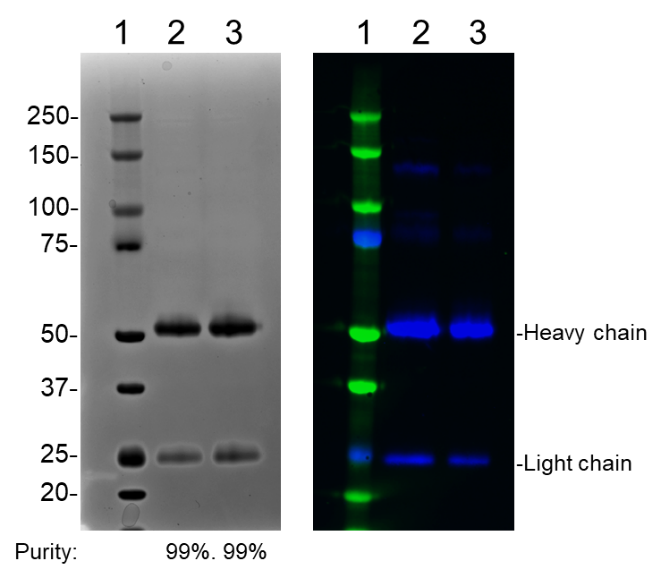


Supplementary Figure 2. SDS-PAGE analysis of purified materials.

IgG purified from sera from ZPIV vaccinees (lane 2) and normal human (lane 3) were analyzed by SDS-PAGE in reduced condition for total protein: Coomassie stain (left) and western blot for human IgG (right). Lane 1 indicates molecular weights (kDa) of the protein marker.

Supplementary Table 1. Summary IgG Purification from ZPIV vaccinees and normal donors

| Donor | Conc. (mg/ml) | Volume (ml) | Total Conc. (mg) | Endotoxin (EU/ml) | ZIKV MN_50_ titer |
| --- | --- | --- | --- | --- | --- |
| ZPIV vaccinees | 47.7 | 7.0 | 333.9 | 0.078 | 16,674 |
| Normal | 47.6 | 9.9 | 471.24 | 0.145 | <10 |

**Characterization of purified polyclonal human immunoglobulin G**

Polyclonal human IgG used for the passive transfer study was prepared as described in Materials and Methods. The quantitative and qualitative characteristics of the purified IgG are indicated in Supplementary Figure 2 and Supplementary Table 1.
