# Supplementary material for: Zika purified inactivated virus (ZPIV) vaccine reduced vertical transmission in pregnant immunocompetent mice
Source: NPJ Vaccines. 2024 Feb 15;9:32. doi: 10.1038/s41541-024-00823-1 (PMC10869681; doi:10.1038/s41541-024-00823-1)
Supplement: Supplementary file 5 — REPORTING SUMMARY [file 41541_2024_823_MOESM5_ESM.pdf]

nature portfolio

Corresponding author(s): In-Jeong Kim

Last updated by author(s): Nov 14, 2023

Reporting Summary

Nature Portfolio wishes to improve the reproducibility of the work that we publish. This form provides structure for consistency and transparency in reporting. For further information on Nature Portfolio policies, see our Editorial Policies and the Editorial Policy Checklist.

Statistics

For all statistical analyses, confirm that the following items are present in the figure legend, table legend, main text, or Methods section.

n/a	Confirmed	
☐	☒	The exact sample size (*n*) for each experimental group/condition, given as a discrete number and unit of measurement
☐	☒	A statement on whether measurements were taken from distinct samples or whether the same sample was measured repeatedly
☐	☒	The statistical test(s) used AND whether they are one- or two-sided<br>*Only common tests should be described solely by name; describe more complex techniques in the Methods section.*
☐	☒	A description of all covariates tested
☒	☐	A description of any assumptions or corrections, such as tests of normality and adjustment for multiple comparisons
☐	☒	A full description of the statistical parameters including central tendency (e.g. means) or other basic estimates (e.g. regression coefficient) AND variation (e.g. standard deviation) or associated estimates of uncertainty (e.g. confidence intervals)
☐	☒	For null hypothesis testing, the test statistic (e.g. *F*, *t*, *r*) with confidence intervals, effect sizes, degrees of freedom and *P* value noted<br>*Give P values as exact values whenever suitable.*
☒	☐	For Bayesian analysis, information on the choice of priors and Markov chain Monte Carlo settings
☒	☐	For hierarchical and complex designs, identification of the appropriate level for tests and full reporting of outcomes
☒	☐	Estimates of effect sizes (e.g. Cohen's *d*, Pearson's *r*), indicating how they were calculated

Our web collection on statistics for biologists contains articles on many of the points above.

Software and code

Policy information about availability of computer code

Data collection	Applied System 7500 Fast System software V1.4.0, Luminex xPonent for MAGPIX v 4.1 build 308, SoftMax Pro V 7.1.2
Data analysis	GraphPad Prism Software v.9.2

For manuscripts utilizing custom algorithms or software that are central to the research but not yet described in published literature, software must be made available to editors and reviewers. We strongly encourage code deposition in a community repository (e.g. GitHub). See the Nature Portfolio guidelines for submitting code & software for further information.

Data

Policy information about availability of data

All manuscripts must include a data availability statement. This statement should provide the following information, where applicable:

- Accession codes, unique identifiers, or web links for publicly available datasets
- A description of any restrictions on data availability
- For clinical datasets or third party data, please ensure that the statement adheres to our policy

All data presented in this publication are available upon request to corresponding authors with the permission by Walter-Reed Army Institute of Research.

Research involving human participants, their data, or biological material

Policy information about studies with human participants or human data. See also policy information about sex, gender (identity/presentation), and sexual orientation and race, ethnicity and racism.

Reporting on sex and gender	N.A.
Reporting on race, ethnicity, or other socially relevant groupings	N.A.
Population characteristics	N.A.
Recruitment	N.A.
Ethics oversight	N.A.

Note that full information on the approval of the study protocol must also be provided in the manuscript.

Field-specific reporting

Please select the one below that is the best fit for your research. If you are not sure, read the appropriate sections before making your selection.

☒ Life sciences ☐ Behavioural & social sciences ☐ Ecological, evolutionary & environmental sciences

For a reference copy of the document with all sections, see nature.com/documents/nr-reporting-summary-flat.pdf

Life sciences study design

All studies must disclose on these points even when the disclosure is negative.

Sample size	Based on the results from the previous publication (Kim et al., 2022), we assigned 10 mice per group to achieve 8 pregnant mice group, which would allow us to detect statistically significant differences in viral burden, antibody titers and other readouts between group. Not all plug detected female mice become truly pregnant at the time of euthanasia of the experimental animals. In two independent experiments, we examined the mock-control (n=3), Alum (n=14) or ZPIV vaccinated (n=15) groups.
Data exclusions	No data were excluded for data analysis.
Replication	Two independent experiments were performed in human STAT 2 knock-in mice with the two-dose vaccination regimen and examined n of 6-8 per group for each of the experiments. For the dose-refining experiments of passive antibody transfer, two independent experiments were performed to test a deescalating dose range from 6 mg to 0.4 mg IgG. For the consistency and reproducibility of data, each of the individual samples was examined in duplicate or triplicate.
Randomization	Five- weeks old female mice were randomly assigned for vaccination prior to pregnancy. After timed mating, gestational day comparable mice were challenged with ZIKV and examined.
Blinding	Sample processing by lab scientists were not blinded to avoid cross contamination between groups.<br>Data collection and analysis were not blinded.

Reporting for specific materials, systems and methods

We require information from authors about some types of materials, experimental systems and methods used in many studies. Here, indicate whether each material, system or method listed is relevant to your study. If you are not sure if a list item applies to your research, read the appropriate section before selecting a response.

Materials & experimental systems

n/a	Involved in the study	
☐	☒	Antibodies
☐	☒	Eukaryotic cell lines
☒	☐	Palaeontology and archaeology
☐	☒	Animals and other organisms
☒	☐	Clinical data
☒	☐	Dual use research of concern
☒	☐	Plants

Methods

n/a	Involved in the study	
☒	☐	ChIP-seq
☒	☐	Flow cytometry
☒	☐	MRI-based neuroimaging

Antibodies

Antibodies used	Purified human IgGs from serum samples obtained from human subjects. Serum samples from human vaccinees were from the RV478 study, "A Phase 1, First-in-human, Double-blinded, Randomized, Placebo-controlled Trial of a Zika Virus Purified Inactivated Vaccine (ZPIV) With alum adjuvant in Healthy Flavivirus-naive and Flavivirus-Primed Subjects". The WRAIR Institutional Review Board (IRB) approved the protocol prior to the study initiation. The trial is registered at ClinicalTrial.gov number: NCT02963909<br><br>Mouse monoclonal pan-flavivirus antibody clone 4G2 were obtained from the commercial vendor, Karafast Inc. the subside of Absolute Antibody Biotech company.
Validation	Information of purified human IgG antibodies is indicated in the Supplementary information.

Eukaryotic cell lines

Policy information about cell lines and Sex and Gender in Research

Cell line source(s)	Green monkey kidney epithelial cells, Vero cells (CCL-81) were purchased from American Tissue and Cell Culture (ATCC)
Authentication	CoA of the Vero cells is avaialble from ATCC
Mycoplasma contamination	Vero cell culture supernatant was tested for Mycoplasma pulmonis and other Mycoplasma species, prior to virus-replication in vitro and the result confirmed to be free of the Mycoplasma.(IMPACT test Case # 24231-2016 by IDEXX BioResearch)
Commonly misidentified lines<br>(See ICLAC register)	N.A.

Animals and other research organisms

Policy information about studies involving animals; ARRIVE guidelines recommended for reporting animal research, and Sex and Gender in Research

Laboratory animals	Breeding pairs of humanized STAT2 knock-in mouse (C57BL/6-Stat2tm1.1(STAT2)Diam/AgsaJ) mice were purchased from JAX mice .<br>The mice were bred and maintained in the animal facility of Trudeau Institute, the accredited by AAALAC.<br>For timed pregnancy study, female mice were used for the study.
Wild animals	N.A.
Reporting on sex	The result reported are applicable for only pregnant females and offspring in response to Zika virus infection.
Field-collected samples	N.A.
Ethics oversight	The mouse studies were conducted following the approved IACUC protocol 19-003 by the Trudeau Institute IACUC committee. All animal studies were approved by by the Animal Care and Use Review Office (ACURO) at the Department of Defense.

Note that full information on the approval of the study protocol must also be provided in the manuscript.

Plants

Seed stocks	N.A.
Novel plant genotypes	N.A.
Authentication	N.A.
